# Gram-Negative Pyomyositis in an Immunocompetent Patient

**DOI:** 10.7759/cureus.2453

**Published:** 2018-04-09

**Authors:** Michelle Knees, Muhammad Talha Ayub, Ajaydas Manikkan

**Affiliations:** 1 Medical Student, Chicago College of Osteopathic Medicine; 2 Internal Medicine, John H. Stroger, Jr. Hospital of Cook County, Chicago, USA

**Keywords:** pyomyositis, gastrocnemius, gram-negative bacteria, klebsiella pneumoniae, immunocompetent

## Abstract

Pyomyositis is an acute or sub-acute primary infection of the striated muscles. It is commonly misdiagnosed in its early stages due to its nonspecific presentation and lower suspicion among physicians when it comes to diagnosis. It has been historically associated with tropical climates but is being seen with increasing incidence in temperate regions. In both tropical and temperate areas, Staphylococcus aureus is the most common causative organism; gram-negative organisms are rare and traditionally only seen in immunocompromised patients. We report a case of Klebsiella pneumoniae pyomyositis in an immunocompetent patient with no risk factors. The awareness of the possibility of gram-negative pyomyositis in immunocompetent patients will broaden initial empiric antibiotic treatment, especially in those patients not responding to traditional empiric treatment.

## Introduction

Pyomyositis is a primary infection of striated muscle. Most cases are reported in immunocompromised patients in tropical areas [1]. Pyomyositis in immunocompetent patients from temperate climates is a documented but rare entity [2]. It presents with non-specific symptoms—commonly fever and muscle pain. If not diagnosed, or if misdiagnosed, pyomyositis has a high morbidity and mortality rate [3]. Empiric treatment in immunocompetent patients is directed against Staphylococcus aureus and streptococci [4]. Gram-negative microbial infections have also been documented, although they are rare [5]. Given the potential for fatal sequelae following undertreated pyomyositis, clinicians should have a lower threshold for starting broader empiric treatment in immunocompetent patients.

## Case presentation

A 59-year-old female presented with a two-week history of progressively worsening right calf pain and swelling that was aggravated with movement and associated with fevers and chills. She denied any prior trauma, insect bites, and knee or ankle pain. She had taken a 12-hour international flight a week before the swelling began.

The patient’s past medical history was only significant for a mitral valve prolapse; she denied any history of hypertension, diabetes, thromboembolic events, or autoimmune disorders. She endorsed smoking five cigarettes per day for the past four years but denied any use of alcohol or illicit drugs.

On admission, she was febrile at 101.3° F with otherwise normal vitals. Her physical exam was unremarkable except for a warm, erythematous, swollen, tender right calf with an overlying maculopapular rash.

**Figure 1:**
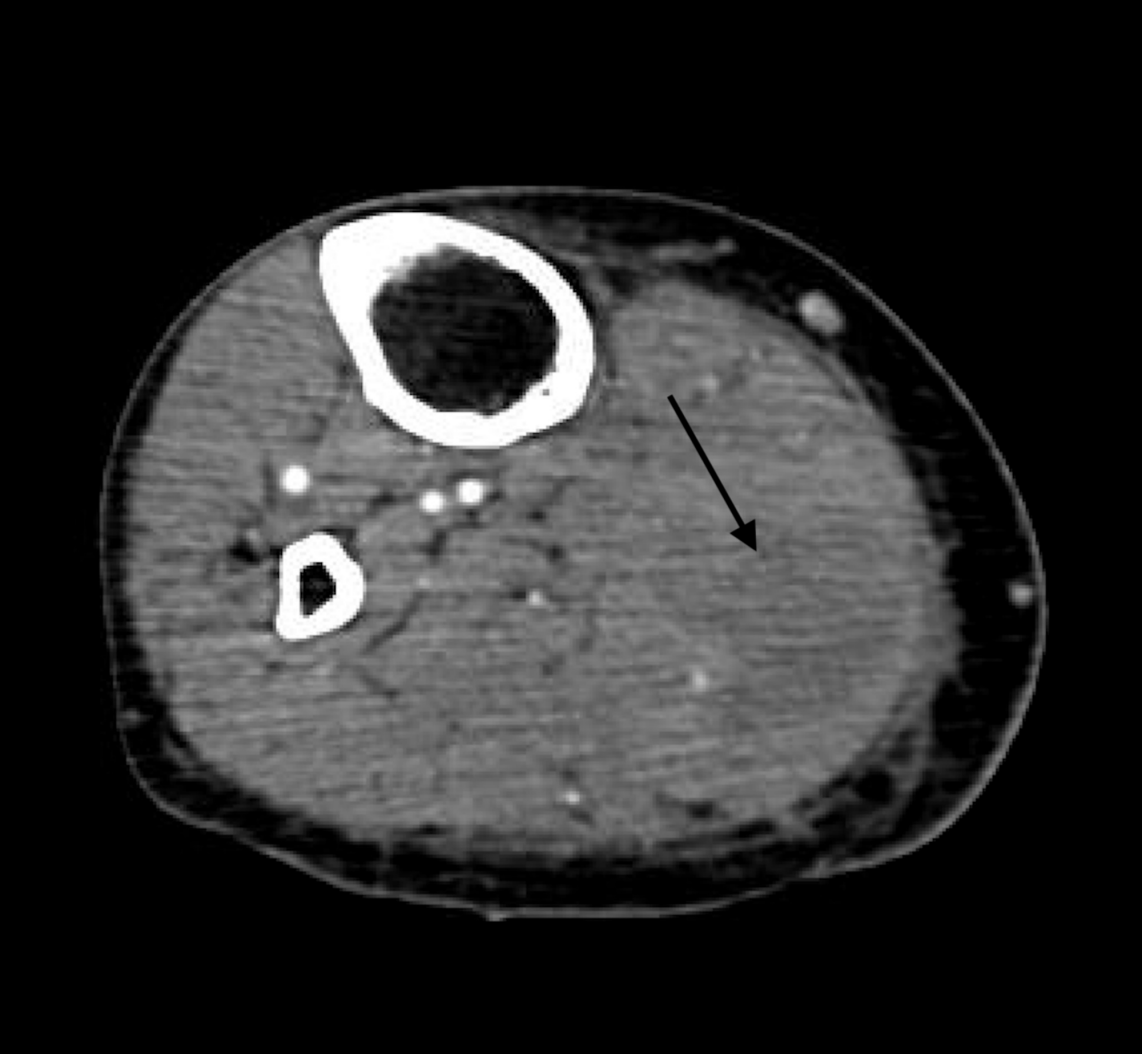
CT angiogram showing fluid collection in gastrocnemius CT: computed tomography

Laboratory results were notable for leukocytosis, elevated erythrocyte sedimentation rate (ESR) and C-reactive protein (CRP), and normocytic anaemia. Urinalysis was normal. A lower extremity ultrasound ruled out deep venous thrombosis. Given the high suspicion for deep vein thrombosis (DVT), lower extremity computed tomography (CT) angiography was done, which revealed a lobulated collection measuring 3.1 cm X 3.8 cm X 10.2 cm within the medial gastrocnemius muscle (Figure 1). Ultrasound-guided drainage yielded 20 ml of purulent fluid, thereby confirming the diagnosis of pyomyositis.

One dose of empiric cefazolin and vancomycin was given in the emergency department after blood cultures were drawn. The extracted fluid was sent for culture, and the patient was treated with ceftriaxone following a second set of blood cultures.

A transthoracic echocardiogram was done next to rule out infective endocarditis as the source; the image results could not definitively rule out vegetations. Transesophageal echocardiography confirmed that there were no vegetations. Human immuno virus (HIV) serology was also negative.

Five days into admission, the wound cultures grew Klebsiella pneumoniae; both blood cultures remained free of growth. The patient made remarkable clinical improvement. She became afebrile, the leukocytosis resolved, and she was discharged with oral trimethoprim-sulfamethoxazole. She was lost to follow-up.

## Discussion

Pyomyositis is a rare bacterial infection, often with associated abscess formation in the striated muscles [1]. While common in tropical climates, it is much rarer in the United States; under 700 cases of pyomyositis have been reported in the literature [6-7]. Pyomyositis is believed to be a primary infection of skeletal muscle occurring after a transient bacteraemia without any contiguous foci from the adjacent skin, soft tissue, or bone [5, 8-9]. Interestingly, it does not usually occur in association with endocarditis or other metastatic infections [9]. Striated muscles are generally resistant to infection and the exact pathogenesis of pyomyositis is still not fully understood [5, 9].

Pyomyositis has classically been a disease process of tropical areas but is gaining recognition as a temperate-climate disease [1-2]. However, pyomyositis in temperate climates is often associated with an immunocompromising condition, most notably HIV. Among those who are HIV negative, nearly half have an immunocompromising condition such as diabetes mellitus, malignancy, or a rheumatological disorder [1]. Pyomyositis has also been reported after trauma, intravenous (IV) drug use, and malnutrition [2].

Staphylococcus aureus is the most common causative microbe, followed by group A streptococci; S. aureus accounts for up to 90% of tropical and 75% of temperate pyomyositis [6, 9]. Gram-negative bacilli are a much rarer cause of pyomyositis; 22 cases have been reported from 1966 through 2000, and five of those were caused by Klebsiella. Unlike our patient, the majority of these patients were immunocompromised or had known risk factors [5].

Pyomyositis traditionally progresses through three stages. Stage one is defined by crampy local muscle pain and a low-grade fever; at this stage, it is treatable with antibiotics alone [9]. However, over 90% of patients will present in stage two with extreme muscle tenderness and a frank abscess. Antibiotics and drainage are typically required at this stage. If untreated or undertreated, patients will progress to stage three, which is defined by extreme muscle pain, fluctuance, and shock [1, 4, 9]. Complications of pyomyositis include septic shock, endocarditis, pneumonia, pericarditis, septic arthritis, brain abscesses, rhabdomyolysis, and death [3, 9]. Given the high morbidity and mortality if untreated, empiric antibiotic treatment should be initiated once the clinician suspects pyomyositis [2, 4, 10]. Traditionally, immunocompetent patients have been treated empirically with broad spectrum antibiotics which cover S. aureus and streptococci until culture-directed therapy can be initiated [4, 10]. However, this case emphasizes that clinicians should have a low threshold for initiating broader empiric treatment to cover for gram-negative species, even in immunocompetent patients.

## Conclusions

Pyomyositis has historically been associated with tropical climates but is now being seen with increasing frequency in temperate areas. When found in an immunocompetent patient, empiric antibiotics which target S. aureus and streptococci are usually initiated; immunocompromised patients traditionally receive broader-spectrum antibiotics which additionally cover gram-negative bacteria. Given the devastating sequelae of undertreated pyomyositis, physicians should consider starting or switching to antibiotics which cover gram-negative bacteria in any immunocompetent patient who is not improving under traditional antibiotics.
